# Resveratrol Attenuates Allergic Asthma and Associated Inflammation in the Lungs Through Regulation of miRNA-34a That Targets FoxP3 in Mice

**DOI:** 10.3389/fimmu.2018.02992

**Published:** 2018-12-20

**Authors:** Esraah Alharris, Hasan Alghetaa, Ratanesh Seth, Saurabh Chatterjee, Narendra P. Singh, Mitzi Nagarkatti, Prakash Nagarkatti

**Affiliations:** ^1^Department of Pathology, Microbiology and Immunology, School of Medicine, University of South Carolina, Columbia, SC, United States; ^2^Environmental Health and Disease Laboratory, Department of Environmental Health Sciences, Arnold School of Public Health, University of South Carolina, Columbia, SC, United States

**Keywords:** Asthma, resveratrol, miRNA-34a, Foxp3, T regulatory cells

## Abstract

Asthma is a chronic inflammatory disease of airways mediated by T-helper 2 (Th2) cells involving complex signaling pathways. Although resveratrol has previously been shown to attenuate allergic asthma, the role of miRNA in this process has not been studied. We investigated the effect of resveratrol on ovalbumin-induced experimental allergic asthma in mice. To that end, BALB/c mice were immunized with ovalbumin (OVA) intraperitoneally followed by oral gavage of vehicle (OVA-veh) or resveratrol (100 mg/kg body) (OVA-res). On day 7, the experimental groups received intranasal challenge of OVA followed by 7 days of additional oral gavage of vehicle or resveratrol. At day 15, all mice were euthanized and bronchioalveolar fluid (BALF), serum and lung infiltrating cells were collected and analyzed. The data showed that resveratrol significantly reduced IL-5, IL-13, and TGF-β in the serum and BALF in mice with OVA-induced asthma. Also, we saw a decrease in CD3+CD4+, CD3+CD8+, and CD4+IL-4+ cells with increase in CD4+CD25+FOXP3+ cells in pulmonary inflammatory cell infiltrate in OVA-res group when compared to OVA-veh. miRNA expression arrays using lung infiltrating cells showed that resveratrol caused significant alterations in miRNA expression, specifically downregulating the expression of miR-34a. Additionally, miR-34a was found to target *FOXP3*, as evidenced by enhanced expression of *FOXP3* in the lung tissue. Also, transfection studies showed that miR-34a inhibitor upregulated *FOXP3* expression while miR-34a-mimic downregulated *FOXP3* expression. The current study suggests that resveratrol attenuates allergic asthma by downregulating miR-34a that induces increased expression of *FOXP3*, a master regulator of Treg development and functions.

## Introduction

Asthma is a chronic inflammatory condition driven by T-helper 2 (Th2)- immune response characterized by cough, shortness of breath and impairment of lung function which might be life threatening (1). The inflammation in asthma leads to increase airway hyper-responsiveness and bronchial spasm and excessive mucous production (2). There are several cellular signaling pathways that are disrupted in asthma resulting in continuous inflammatory response often to harmless substances such as dendrites and pollen (3). One of the disrupted pathways, most often found, comprises of changing the expression of co-stimulatory molecules and cytokines secretion (4). As a Th2-driven inflammatory response, asthma is characterized by increase in the levels of IL-4, IL-5, IL-13, and GM-CSF along with an increase in the level of TNF-α and TGF-β in BALF and serum, which primarily act as pro-inflammatory cytokines (5). It is well-known that asthma and autoimmune diseases with impaired peripheral tolerance are associated with T-regulatory cell dysfunction (6). T-regulatory (T-regs) cells are subpopulation of T-cells responsible for dampening the immune response, maintenance of peripheral tolerance and prevention of chronic inflammation (7). T-regs are known to express CD4 and CD25 surface molecules in addition to secretion of anti-inflammatory cytokines represented by IL-10 (8). The most important transcription factor for T-reg is *FOXP3*, which plays an important role in their differentiation and development (9). In fact, the knockdown of *FOXP3* results in development of autoimmune diseases in multiple organs due to absence of properly functioning T-regs (10). Tregs have been found to be defective in both number and function in allergic disease and their mutation has been linked to exaggerated immune response (11–15).

micro-RNAs (miRNAs) are small, ~22 nucleotide long, non-coding, regulatory RNAs that play a critical role in the regulation of gene expression at the post-transcriptional level. miRNAs play a significant role in driving asthma-related immune responses, although the exact role and subsequent downstream pathways of regulation remain obscure. Significant alterations in the expression of miRNA in airway epithelial cell have been reported in asthma (16). These alterations are only modestly corrected by inhaled corticosteroids (16), thereby showing that alternative modes of treatment are critical that could alter miRNA expression.

Resveratrol is a poly-phenolic stilbene (3, 4, 5-trihydroxystilbene) which is abundant in wine, skin of red grapes, berries, and peanuts (10, 17, 18). Several previous studies demonstrated that resveratrol has anti-carcinogenic, anti-inflammatory and cardio-protective effects (10, 19, 20). Asthma is one of the diseases in which T-regs function is impaired, and the response of asthmatic patient to systemic or inhaled steroid is attributed mainly to their ability to induce immunosuppression via T-reg cell activation (6). The non-availability of a specific drug to target the downstream pathways of Th2-driven mechanisms and their modulations by miRNAs restricts therapy in these conditions (21). The situation is also complicated by an overdependence on long acting corticosteroids, which has potential side effects (22–24). We and others have shown that resveratrol is highly effective in suppressing inflammation in many autoimmune disease models (25–27). Furthermore, researchers have shown that resveratrol is as effective as steroids in treatment of asthma (28, 29). However, the role of miRNA in resveratrol-mediated attenuation of allergic asthma, its ability to induce the expression of *FOXP3*, a master regulator of Tregs and immunosuppression, has not been previously investigated. In the current study, we used a murine model of asthma to study the therapeutic effects of resveratrol and to address the role of miRNA. Our results showed that resveratrol induces the repression of miR34a leading to over expression of *FOXP3* as a possible mechanism of attenuation of allergy/asthma symptoms in mice.

## Materials and Methods

### Animals

Female BALB/c mice aged 6–8 weeks were purchased from Jackson laboratories. All mice were housed in specific pathogen-free conditions at the AAALAC-accredited animal facility at the University of South Carolina, School of Medicine (Columbia, SC). All performed experimental procedures were approved by the University of South Carolina Institutional Care and Use Committee (IACUC). The mice were maintained under a 12 h light/dark cycle at an ambient temperature of 24 ± 1°C in a specific pathogen-free animal facility. They received *ad libitum* access to normal chow diet and water. Cages were cleaned every other day and cocoon enrichment was renewed at the same time. All the experiments were performed in accordance with the University of South Carolina IACUC approved protocol AUP2377. Sample size was estimated based on power analysis and we used groups of 3–5 mice as detailed in the Figure legends. The mice were kept in the facility for at least a week for acclimatization before use in the experiments.

### Effect of Resveratrol on Asthmatic Lungs

Because previous studies have shown that female BALB/c mice are more susceptible to asthma (30–32), we used these mice in this study. The mice were divided randomly into three groups (Naïve, OVA-vehicle was designated as OVA-veh, and OVA-resveratrol was designated as OVA-res) in isolated cages. The mice from the Naïve group did not get any treatment whereas, mice from the other two treatment groups were first sensitized intraperitoneally with 250 μg of chicken egg-derived ovalbumin (OVA), dissolved in a solution of aluminum hydroxide (4 mg/ml) in a sterile phosphate buffer saline without Ca++ and Mg++ (PBS), and this day was designated as day 0 of the study (33). On day 7, both sensitized groups of mice were challenged with 50 μg OVA suspended in 50 μl of sterile PBS intranasally under short anesthesia. The OVA-res group were administered by oral gavage, 100 mg/kg resveratrol dissolved in carboxyl methyl cellulose (CMC) in volume of 200 μl daily from day 1 through day 14 of the experiment (17, 34–36). We chose the dose (100 mg/kg body weight) of resveratrol based on our previous studies in which we found this dose to be optimum (17, 34–36). The mice of vehicle treatment group (OVA-veh) were given CMC (200 μl) by oral gavage. All the mice were euthanized on day 15 under anesthesia.

### Histopathology of the Lung

After euthanasia, the lungs were harvested and then perfused with 4% paraformaldehyde in 0.1 M PBS. After perfusion, harvested lung tissues were first fixed in formalin solution (10%) and then were embedded in paraffin blocks. Paraffin section of lung tissue were cut using a microtome to a thickness of (5 μm). The lung section on slides were stained with Hematoxylin and Eosin (H&E) dyes. H&E stained lung tissue section were assessed for histological changes and the cellular infiltration into the lung tissues were analyzed using Leica DM 2500 fluorescent microscope (Buffalo Grove, IL, USA).

### Analysis of Cytokines

The generation of cytokines post vehicle or resveratrol treatment was analyzed in the bronchoalveolar lavage fluid (BALF) and the serum. The analysis of cytokines in BALF was performed using the protocol as described previously (37). Briefly, trachea was first tightened using suture and then lungs were isolated as an intact organ with sutured trachea. Then, sterile ice-cold PBS (1 ml) was injected into the trachea and the draining fluid (BALF) was collected. The collected BALF was centrifuged at 300 × g for 15 min and the supernatant was collected and used for cytokines analysis. To analyze cytokines in sera, blood was collected on day 15 from the retro-orbital space under light anesthesia. The serum was isolated by centrifugation at 300 × g for 15 min at 4°C. The collected supernatant was used for cytokine analysis by ELISA.

Cytokines in collected BALF and sera were detected using sandwich enzyme-linked immunosorbent assay (ELISA) kits for IL-5 and IL-13, obtained from Affymetrix (Santa Carla, CA, USA) and free-TGF-β ELISA kit, obtained from Biolegend (San Diego, CA, USA). The protocols of the manufacturer were used to perform ELISA. The absorbance values were measured by Vector2 microplate reader from PerkinElmer (Boston, MA).

### Analysis of Lung Infiltrating by Flow Cytometry

Lung infiltrating cells were analyzed by flow cytometry. Lung infiltrating cells were isolated using Histopaque and following the protocol as described earlier (38). In brief, lungs were first perfused with cold PBS and then harvested from various treatment groups and single cell suspensions were prepared as described earlier (39, 40). RBC lysis buffer from Biolegend (San Diego, USA) was added (250 μl/lung) for 1 min to lyze the red blood cells. FACS buffer was then added and the cells were collected by centrifuging at 300 × g for 10 min at 4°C. Next, the cells were suspended in 5 ml of FACS buffer and then Histopaque (at room temperature) was added. The Histopaque columns containing cells were centrifuged at 500 × g for 30 min at room temperature. The cells present in the interphase layer were carefully collected Nd transferred to another 15 ml tube containing 10 ml of PBS. The cells were then washed twice in complete medium (Dulbecco's Modified Eagle Medium (DMEM) supplemented with 10% Fetal Bovine Serum (FBS), 1% penicillin/streptomycin). The collected cells were suspended in complete medium and the cells were counted using automated cell counter from Bio Rad (Hercules, CA, USA). The cells were then stained using fluorophore labeled surface markers. Staining with antibodies against intracellular cytokine was performed using Fixation/Permeabilization Solution Kit (BD, San Jose, CA, USA). Probing the intranuclear transcription factors was done using True-nuclear Transcription Factor Buffer set (Biolegend, San Diego, CA, USA). The following antibodies directed against mouse markers: PE-anti-CD3 conjugated with PE or PE/cy5.5 fluorophore, anti-CD4 conjugated with PE/cy7, FITC or APC fluorophore, anti-CD8 conjugated with FITC, anti-IL-4 conjugated with PE/cy7 fluorophore, anti-CD25 conjugated BV-786, and anti-FOXP3 conjugated with PE. CD25+FOXP3+ cell percentage was gated on CD3+CD4+ cells. The stained cells were then analyzed using BD-FACS Celesta flow cytometer and their DIVA software (BD Biosciences, San Diego, CA). The total cell number of CD3+CD4+ and CD3+CD8+ was calculated and expressed by multiplying the percentage of positive cells by the total cell number divided by 100.

### miRNAs Array

For miRNAs arrays, total RNAs including miRNAs isolated from lung infiltrating cells were used. We had analyzed three samples each of OVA+veh and OVA+res groups and two samples of Naïve group. The reason we did not analyze three Naïve samples is that other coworker in the lab had analyzed five Naïve samples. We selected the three samples randomly from five samples to perform miRNAs array assay. In brief, total RNA from lung infiltrating cells was isolated using RNA assay kit from Qiagen (Valencia, MD, USA) and following the protocol of the company. Total RNA was then labeled using kit from Qiagen and following the protocol of the company for labeling. Briefly, the volume and the concentration of RNAs from various samples were adjusted and then control oligonucleotides were added. Ligase was added to ligate the RNAs end to biotin. The labeled miRNAs from various samples were added to the cartridges and hybridization was performed. After 18 h of hybridization, the cartridges were washed and stained using GCS3000 System from Affymetrix (Santa Carla, CA, USA) and following the manufacturer's protocols. The cartridges were then scanned using Affymetrix scanner. The results were analyzed using Expression Console software from Affymetrix. The dysregulated miRNAs were then analyzed using Ingenuity Pathway Analysis (IPA) software from Qiagen. To identify the target genes, we uploaded only those miRNAs demonstrating a fold change ≥2 or ≤ -2 into IPA software. Next, we used the website, http://www.microrna.org/, to show the alignment between miRNA and the target genes.

### Quantitative Real-Time PCR (qPCR) to Validate miRNAs and Genes Expression

To validate the expression of miRNAs and genes of interest, total RNAs isolated from pulmonary infiltrating mononuclear cells from the lungs of Naïve, OVA-veh, and OVA-res groups were used. The concentration and the quality of RNAs were analyzed using Nanodrop 2000 (Thermo Fisher Scientific, Rockford, IL, USA). Equal amounts of RNAs were used for cDNA synthesis. To examine the expression of miRNAs, cDNAs were synthesized using miScript cDNA Synthesis kit and following the protocol of the company (Qiagen, Valencia, MD, USA). SYBR Green PCR kit from Qiagen was used and the protocol of the company was followed. To detect gene expression, SSO- Advanced SYBR Green PCR kit (Bio-Rad, Hercules, CA) was used and the protocol of the company was followed. Real-Time PCR was performed for 39 cycles and the details are as follows: 30 s 98°C (denaturation step), 60 s at 60°C (annealing step) and 60 s at 72°C (extension step, followed by incubation for 10 min at 72°C. The gene expression was normalized to GAPDH. GAPDH housekeeping gene was used because its expression is reliable for one kind of cells 41, 42. The PCR results of miRNAs expression were normalized to Snord 96A (small nucleolar RNA, C/D box 96A) was used as a control to assess the level of miRNA (43). The details of miRNAs and primer sequences for genes used in qPCR are described in Supplementary Table 1.

### Immunofluorescence and Immunohistochemistry Assays

To validate the PCR results of upregulated *FOXP3* gene expression, we performed immunofluorescent staining of lung tissue slides. We calculated the corrected total cell fluorescence (CTCF) for FOXP3-stained cells in the lung tissue of naïve, OVA-veh, and OVA-res group using ImageJ software from NIH, we performed immunofluorescent staining as described earlier (44). The slides with section of lung tissue were first deparaffinized according to the standard protocol, antigen retrieval was done using antigen-retrieval solution from Abcam (Cambridge, MA, USA). The slides were then washed with PBS twice, permeabilized with 0.01% Triton X-100 (Sigma) for 15 min, washed three times with PBS 5 min each, and then incubated overnight at 4°C with primary antibody (mouse-specific FOXP3) antibody from Abcam (Cambridge, MA, USA) diluted in 1% FBS in PBS. Next, the slides were washed with PBS three times 10 minutes each and incubated with anti-mouse secondary antibody diluted in 1% FBS in PBS for 1 h at 37°C followed by washing the slides three times with PBS. The slides were then stained with DAPI to show the nuclei of the cells and washed three times with PBS and finally mounted with Antifade Mounting Medium from Vector Labs (Burlingame, CA). The tissue was visualized and photographed using Leica Fluorescent microscope.

For immunohistochemistry analysis, the lung tissues section (5 μm thickness) were first deparaffinized using a standard protocol. Epitopes were retrieved using epitope retrieval solution and steamer (IHC-Word, Woodstock, MD). Endogenous peroxidases were blocked using 3% H_2_O_2_ for 10 min in dark followed by serum blocking. The tissue was incubated with primary antibody anti-FOXP3 (Abcam, Cambridge, MA, USA) at 4°C overnight. Species-specific biotinylated secondary antibody and streptavidin conjugated with HRP (Abcam) were used to implement antigen-specific immunohistochemistry. The chromogenic substrate 3,3'-Diaminobenzidine (DAB) (Sigma Aldrich, St Louis, MD) was used followed by Mayer's Hematoxylin solution (Sigma Aldrich) as a counterstain. Section were washed between the steps using phosphate buffered saline 1X. Finally, stained section were mounted with Simpo-mount (GBI laboratories, Mukilteo, WA). Tissue section were observed using Olympus BX51 microscope (Olympus, America). Image Pro Plus software from Media Cybernetics (Rockville, MD) was used for morphometric analysis of images.

### Transfection of Splenocytes With miR-34a

Splenocytes were collected from naïve female BALB/c mice and cultured in complete RPMI (Roswell Park Memorial Institute) medium supplemented with 10% FBS and 1% penicillin/streptomycin. The cells were seeded at density of 2 × 10^5^ cells /well in a 24-well plate and were activated with 1 μg/ml SEB overnight (39). The cells were then transfected either with 20 nmol mock control obtained from Qiagen or with miR-34a mimic or anti-miR-34a (inhibitor) using HiPerfect Transfection reagent from Qiagen. The transfected cells were cultured for 24–48 h. The transfected cells were then collected and used for total RNAs including miRNAs isolation. Total RNAs were then used for validation of miR-34a and FOXP3 gene expression.

### Statistical Analysis

We used groups of five mice to study the role of resveratrol in OVA-induced allergy. One-way ANOVA with *post-hoc* Tukey's test was used to compare between three groups. Student's *t*-test was used to compare two groups. In all experiments, data were shown as mean ± S.E.M. and *p* <0.05 was regarded statistically significant.

## Results

### Resveratrol Treatment Improves Th2-Mediated Immune Response

**Figure 1:**
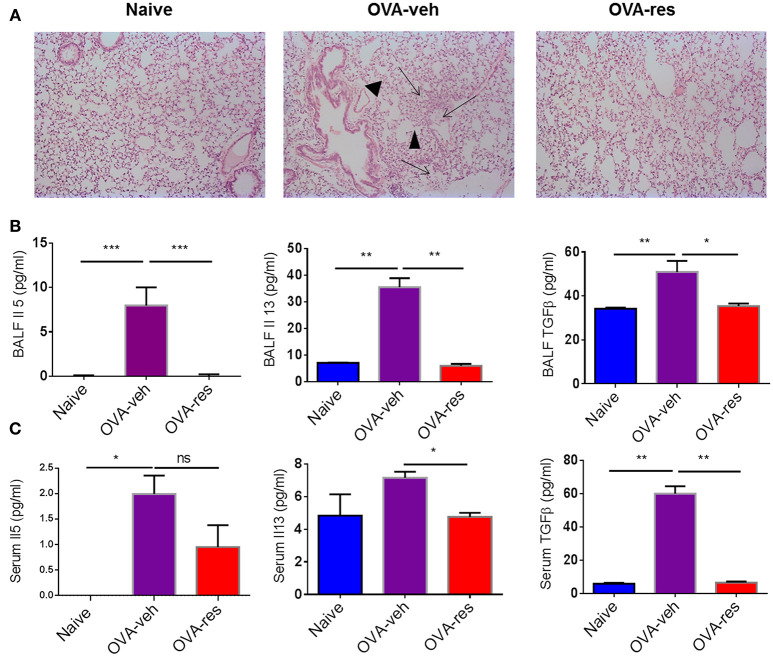
Resveratrol attenuates Ova-induced asthma. As detailed in Methods, mice were administered Ovalbumin (OVA) intraperitoneally followed by intranasal challenge and treated with vehicle (OVA-veh) or 100 mg/kg Resveratrol (OVA-res) daily by oral gavage for 2 weeks and sacrificed on day 15. **(A)** Histopathological analysis of the lungs. Significant thickening was observed in the alveolar walls and inflammatory cell infiltration as well as accumulation of edematous fluid in the alveolar spaces (arrows) and destruction of airways (arrowheads) in OVA-veh group when compared to naïve or OVA-res groups. Measurements of asthma-associated cytokines are shown for BALF **(B)** and serum **(C)**. The number mice used are given in parenthesis: Naïve (*n* = 5), OVA-veh (*n* = 3), and OVA-res (*n* = 3). Asterisks (^*^) represent significant (*p* < 0.05) difference for all experiments. ANOVA and *post-hoc* Tukey's tests were performed to determine the significant (^*^p < 0.05, ^**^p < 0.01, ^***^p < 0.001) difference between the groups.

Hematoxylin and eosin staining of the lung tissue was assessed by blind observer. The examination revealed that the lung tissue of OVA-veh group was congested due to presence of perivascular and perialveolar inflammatory cell infiltrate and fluid extravasation along with destruction of alveolar walls compared to naïve group. Treatment with resveratrol resulted in restoration of lung tissue architecture, reduction in the inflammatory cell infiltrate, and disappearance of inflammatory exudate. The parenchyma of the treated group (OVA-res) was comparable to the naïve group with normal alveolar wall, clearance of inflammatory cell infiltrate and reduced alveolar wall edema (Figure 1A).

**Figure 2:**
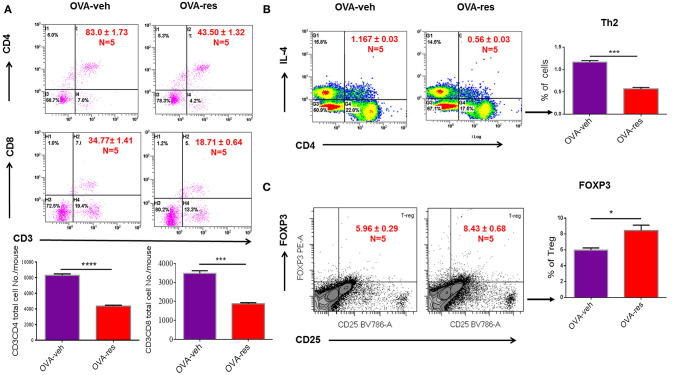
Resveratrol decreases T cell subpopulations in lungs of OVA-administered mice. Resveratrol was used to treat OVA-induced asthma as described in Figure 1. Lung infiltrating inflammatory cells were stained by flow cytometry. **(A)** Shows a representative flow cytometric analysis of CD3+CD4+ and CD3+CD8+ T cells. Data presented in percentage in each Flow data represent the value of three independent experiments. Vertical bars represent total cell number/mouse (Mean+/– SEM) from multiple experiments. **(B)** Shows a representative flow cytometric analysis of CD4+IL4+ Th2 cells. **(C)** Shows a representative flow cytometric analysis of CD25+FoxP3+ Tregs gated on CD3+CD4+ cells. For **(B,C)**, vertical bars represent percentage of cells/mouse (Mean+/– SEM). The number mice used are given in parenthesis: Naïve (*n* = 5), OVA-veh (*n* = 5), and OVA-res (*n* = 5). Asterisks (^*^) represent significant (*p* < 0.05) difference between two groups using Student's *t*-tests (*p* < 0.05).

OVA-veh group showed a significant increase in the levels of IL-5, IL-13, and free active TGF-β in BALF when compared to the naïve group while these cytokines were significantly lower in BALF of OVA-res group when compared to OVA-veh (Figure 1B). Furthermore, the level of serum IL-13, and TGF-β but not IL-5, were significantly lower in the OVA-res group when compared to OVA-veh (Figure 1C). When we analyzed T cells in the lungs, we noted that the total cell count of CD3+CD4+ and CD3+CD8+ was significantly reduced in OVA-res group when compared to OVA-veh group (Figure 2). Also, percentage of cells expressing CD3+CD4+IL-4 (Th2 cells) was significantly lower in OVA-res group when compared to OVA-veh (Figure 2B). Furthermore, we looked for percentage of cells expressing CD25+FOXP3+ (Tregs) in the lung infiltrating cells and found that there was significant increase in the percentage of Tregs in resveratrol treated group when compared to vehicle (Figure 2C).

### Treatment With Resveratrol Alters the miRNAs Profile

**Figure 3:**
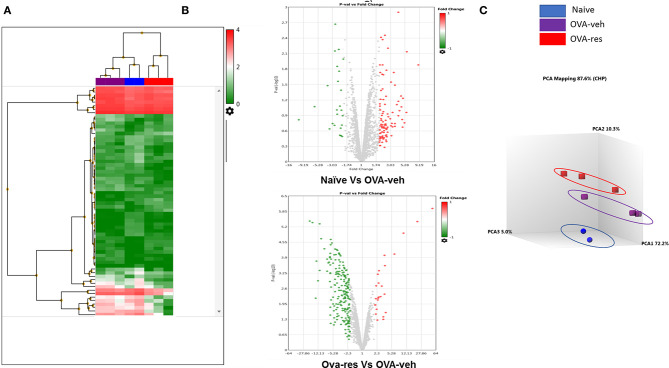
Resveratrol treatment alters miRNA profile in lung infiltrating cells. OVA-induced asthmatic mice were treated with resveratrol as described in Figure 1. Lung infiltrating cells from naïve, OVA-veh, and OVA-res groups were isolated and total RNAs including miRNAs were used to perform miRNA arrays. **(A)** Heat map showing miRNAs expression profile with red representing upregulated while blue representing downregulated miRNAs. **(B)** Volcano Plot shows that OVA-res group has higher levels of downregulated miRNAs when compared to OVA-veh group. **(C)** Principal component analysis (PCA) plot showing separation of miRNA in the three groups. The number of mice used (Naïve: *n* = 3, OVA-veh: *n* = 3, and OVA-res: n=3).

Transcriptome Analysis-generated heat map and volcano plot analysis showed that most of the altered miRNA were downregulated (Figures 3A,B). Principal Component Analysis of two independent samples showed distinct clustering of miRNA profiles of naïve, OVA-veh and OVA-res groups (Figure 3C).

### Resveratrol Treatment Leads to Alterations in miRNAs That Target *FOXP3, IL-13, IL-10*, and *GATA-3*.

**Figure 4:**
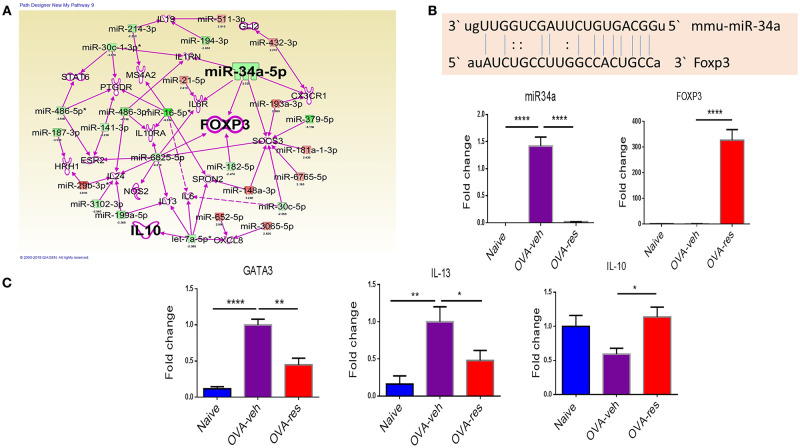
Analysis of dysregulated miRNAs and regulation of *Foxp3* and cytokine genes post resveratrol treatment. **(A)** Ingenuity Pathway analysis was performed to analyze dysregulated miRNAs and identify target molecules. **(B)** Shows the alignment of targeted gene and miR-34a using miRNA.org software. qPCR was performed to validate of the expression of miR-34a **(C)** and its target gene FOXP3 **(D)**, transcription factor GATA3 **(E)**, cytokine IL-13 **(F)**, and cytokine IL-10 **(G)**. The number of mice used (Naïve: *n* = 5, OVA-veh: *n* = 5, and OVA-res: *n* = 5). Data are shown as Mean +/– SEM of triplicates and asterisks (^*^) represent significance (^*^p < 0.05, ^**^p < 0.01). Significance of data was determined using ANOVA test and *post-hoc* Tukeys test. Each experiment was repeated at least three times.

IPA analysis led to identification of anti-inflammatory pathways such as *FOXP3* (transcription factor for T-regulatory cells) and *IL-10* (Figure 4A) as potential targets of miRNA. In this analysis, we also identified that miR-34a may target *FOXP3* gene (Figure 4A), which was confirmed by gene alignment software (Figure 4B). Real-time qPCR analysis in lung infiltrating cells validated the findings that miR-34a was induced while *FOXP3* was suppressed in OVA-veh group and treatment with resveratrol led to significant downregulation miR-34a while increasing the levels of *FOXP3* (Figure 4B). We also studied in these cells, genes related to Th2 such as *GATA-3* (Th2-transcription factor) and *IL-13* (Th2-related cytokine) and both were significantly downregulated in OVA-res when compared to OVA-veh (Figure 4B). Interestingly, *IL-10* gene expression was also significantly higher in OVA-res when compared to OVA-veh (Figure 4C).

### Resveratrol Treatment Leads to Induction of Foxp3+ Cells in the Lung

**Figure 5:**
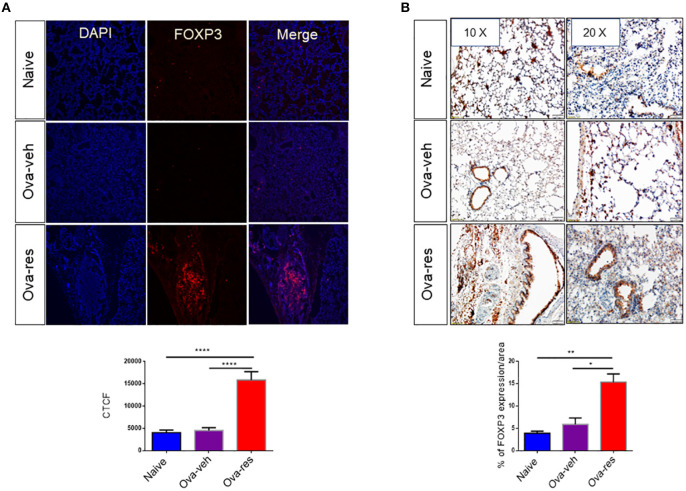
Resveratrol treatment leads to induction of *FOXP3*+ cells in the lungs: Immunofluorescence and immunohistochemistry were performed to determine the expression of *FOXP3* in lung tissues and FoxP3 expression in the cells was assessed using corrected total cell fluorescence (CTCF) and ImageJ software. **(A)** Shows the expression of *FOXP3* in lung tissues. The data in vertical bars represent Mean+/– SEM of 10 random spots analyzed. Significance (^*^p < 0.05) of FoxP3 expression between the groups was analyzed using Student's *t*-test. **(B)** Shows FoxP3 expression in lung tissues by performing immunohistochemistry. The data represented as Mean+/– SEM of random 3–5 spots that were analyzed. The number of mice used (Naïve: *n* = 3, OVA-veh: *n* = 3, and OVA-res: *n* = 3). Significance (^**^p < 0.01, ^****^p < 0.0001) in FoxP3 expression was detected using one-way ANOVA and *post-hoc* Tukey's test.

We found that corrected total cell florescence (CTCF) for FOXP3 protein was significantly higher in OVA-res group when compared to naïve and OVA-veh group (Figure 5A). Moreover, immunohistochemistry staining of lung tissue section also showed significant increase in the levels of FOXP3 expression in OVA-res group when compared to OVA-veh (Figure 5B).

**Figure 6:**
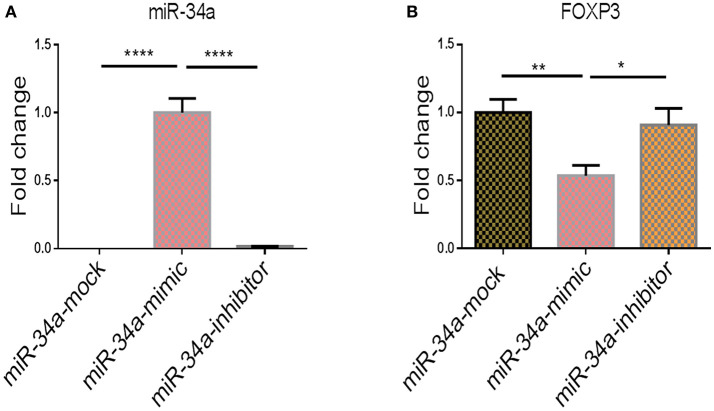
The effect of transfection of miR-34a mimic and inhibitor on *Foxp3* gene expression. qPCRs were performed to determine the expression of miR-34a and FoxP3 post transfection of splenocytes with miR-34a mimic and miR-34 inhibitor. **(A)** Shows the expression of miR-34a and **(B)** shows the expression of FoxP3 in splenocytes post transfection. The data presented as vertical bars represent Mean+/– SEM of triplicates. Significance (^*^p < 0.05, ^**^p <0.01, ^****^p <0.0001) of miR-34a and FoxP3 expression was determined using ANOVA test and Tukey's-hoc test.

To show further corroborate that the overexpression of *FOXP3* was due to downregulation of miR-34a, we performed transfection studies using mock, miR-34a mimic or inhibitor in splenocytes that were activated with SEB. We observed that upregulation of miR-34a using a mimic, was associated with downregulation of *FOXP3*, while use of miR-34a inhibitor led to upregulation of *FOXP3* gene (Figure 6). Together, these data demonstrate that miR-34a was targeting *FOXP3*.

## Discussion

Asthma is a common reason for morbidity in children and adults caused by chronic inflammatory response mediated by Th2-immune response (45–47). Although a wide range of pharmacological therapies have been developed as a prophylactic treatment or to control acute asthmatic attacks such as β2-adrenergic drugs, steroids, IgE blockers, methylxanthines, and leukotriene modifying agents, the most effective treatment stems from the use of steroids (48, 49). However, steroids are associated with serious systemic side effects such as osteoporosis, cataract, growth retardation in children and immunosuppression (50). Resveratrol, a polyphenolic stilbene is effective against asthma (50, 51) without the serious side effects as shown by many randomized clinical trials (52–54). In the current study we demonstrated that resveratrol can efficiently attenuate allergic asthma in a mouse model. These data are consistent with other studies demonstrating that resveratrol can suppress both airway inflammation and airway structural changes in mouse models of bronchial allergic asthma (28, 51, 55, 56). It is noteworthy that while such studies have identified many signaling pathways through which resveratrol can attenuate allergic asthma including suppression of expression of TGF-β1/phosphorylated Smad2/3 (28), inhibition of Syk protein expression (55), and increased expression of INPP4A in lungs which in turn reduced Akt kinase activity and Akt phosphorylation (51), the potential role played by miRNA in resveratrol-mediated attenuation of allergic asthma has not been previously investigated. In this study, we found that resveratrol treatment of ovalbumin-induced asthma in mice was associated with downregulation of several miRNA, particularly, miR-34a which targeted *FOXP3* gene, a T-reg transcription factor, and caused significant induction of this gene, thereby potentially inhibiting the Th2-mediated immune response.

The present study also showed that resveratrol treatment significantly reduced the levels of Th-2 cells and Th2-related cytokines (IL-5, and IL-13) in addition to asthma-related cytokines such as TGF-β in BALF, and serum. The reduction in the level of asthma-related cytokines is an important indicator of suppression of inflammatory response (57, 58). On the other hand, the level of IL-10 gene expression, encodes for an anti-inflammatory cytokine primarily secreted by T-regs, was also significantly higher in the pulmonary cell infiltrate from OVA-res group when compared to OVA-veh-treated group. This finding indicates that resveratrol may promote IL-10 mediated immune resolution as reported previously (59, 60). These data on suppression of inflammation were consistent with our observation that the levels of CD3+CD4+, CD4+IL4+(Th2), and CD3+CD8+ was significantly reduced in Resveratrol treated group as compared to the asthma controls (OVA-veh).

It has been shown previously that miRNA play an important role in regulation of inflammation. For example, in the innate immune system, miR-155 was shown to mediate the effect of LPS (lipopolysaccharide) on macrophages via TLR (Toll-Like Receptor) signaling pathway (61). In addition, inflammatory response to infection in macrophages was mediated by upregulation of many miRNAs like miR-9, miR-21, and miR-149 (62–64). The adaptive immune system was also proven to be regulated by miRNA alteration. Differentiation of naïve T-cell to effector T- cell was reported to be driven by miRNA regulation (65, 66). In addition, miRNAs also regulate B-cell differentiation, maturation and activation (67–69). Even though resveratrol can alter the miRNA, very few studies mention the role of miRNA in resveratrol-mediated anti-inflammatory effect in Ovalbumin-induced asthma (70–76). In the current study, we found that miR-34a was significantly downregulated in OVA-res group as compared to OVA-veh. Moreover, we found that miR-34a showed good alignment with *FOXP3* gene using IPA analysis and its subsequent downregulation by resveratrol treatment might significantly upregulate *FOXP3*. Following the same cues, we performed further studies to mimic or inhibit the expression of miR-34a by transfecting the cell with Hi-perfect miR-34a mimic or inhibitor and compared that with mock control. The results showed that the over-expression of miR-34a significantly downregulated *FOXP3* gene and the administration of the mir-34a inhibitor significantly upregulated *FOXP3* gene when compared to mock control. Taken together, these findings indicated that anti-inflammatory effect of resveratrol in ovalbumin induced asthma may be mediated by downregulation of miR-34a which in turn upregulated *FOXP3*.

It has been shown previously that miRNA play a central role in the induction of Tregs (77). For example, deletion of miRNAs by lineage-specific ablation of Dicer or Drosha in T cells was found to decrease the number of Tregs, leading to fatal multiorgan inflammatory disease (78). Also, miR-155 knock out (KO) mice have reduced numbers of *FOXP3*+ cells (79). Since such reports, many studies have identified other miRs such as miR-10b, miR-99a, miR-130a, miR-146b, miR-150, and miR-320 that can drive Treg differentiation (77). While the role of miR-34a in regulating *FOXP3* expression and Treg induction has not been previously reported, we found one study in which lipopolysaccharide suppressed miR-34a leading to upregulation of CCL2, a macrophage-derived chemokine that recruits Tregs (80).

In the current study, we also noted that OVA-sensitized mice showed higher levels of TGF-β and resveratrol treatment led to decrease in this cytokine production. Interestingly, TGF-β plays many roles. In asthma, TGF-β acts as a major mediator involved in pro-inflammatory responses and fibrotic tissue remodeling within the asthmatic lung (81). It is also known to promote Th2 cytokine profile and elevated TGF-β levels have been detected in asthmatic airway (81). These observations are consistent with our current study in which we found that TGF-β levels were upregulated in OVA-exposed mice and that treatment with resveratrol led to significant decrease in TGF-β levels. Thus, resveratrol may also help suppress lung inflammation by targeting TGF-β. TGF-β is also known for its Yin-Yang role and thus, it can also induce Tregs (82). However, in our model, it is more likely that the Treg induction by resveratrol may stem from its action on miR-34a.

The limitation of this study was using female BALB/C mice which is due to the fact that they are more susceptible to get allergic airway diseases than males (30–32). In addition, we need to validate the results by doing *in-vivo* transfection with miR-34a mimic and inhibitor and look at the ability of them to develop allergic airway diseases.

In summary, our study showed that oral administration of resveratrol suppressed the asthma-associated immune response and its action was mediated by upregulation of *FOXP3* induced by miR-34a down-regulation. To the best of our knowledge, we report a novel finding that resveratrol improves asthma via its effect on *FOXP3* expression in pulmonary infiltrating cell and its therapeutic effect is mediated by miR-34a inhibition. This study will go a long way in establishing therapeutic strategies in treatment of asthma without the anti-asthmatics-associated side effects.

## Author Contributions

MN, PN, and EA conceptualized the work. MN and PN provided all the resources for the study. EA carried out all experiments while HA helped in some experiments. RS and SC helped pursue studies on *FOXP3* detection in lung tissue. NS helped in discussion and edited the manuscript.

### Conflict of Interest Statement

The authors declare that the research was conducted in the absence of any commercial or financial relationships that could be construed as a potential conflict of interest.

## References

[1] Barnes PJ. Immunology of asthma and chronic obstructive pulmonary disease. Nat Rev Immunol. (2008) 8:183–92. 10.1038/nri2254 18274560

[2] Howarth PH Bradding P Montefort S Peroni D Djukanovic R Carroll MP. Mucosal inflammation and asthma. Am J Respir Crit Care Med. (1994) 150(5 Pt 2):S18–22. 10.1164/ajrccm/150.5_Pt_2.S18 7952584

[3] Spellberg B Edwards JE Jr. Type 1/Type 2 immunity in infectious diseases. Clin Infect Dis. (2001) 32:76–102. 10.1086/317537 11118387

[4] Chakir J Shannon J Molet S Fukakusa M Elias J Laviolette M. Airway remodeling-associated mediators in moderate to severe asthma: effect of steroids on TGF-beta, IL-11, IL-17, and type I and type III collagen expression. J Allergy Clin Immunol. (2003) 111:1293–8. 10.1067/mai.2003.1557 12789232

[5] Karagiannidis C Akdis M Holopainen P Woolley NJ Hense G Rückert B. Glucocorticoids upregulate FOXP3 expression and regulatory T cells in asthma. J Allergy Clin Immunol. (2004) 114:1425–33. 10.1016/j.jaci.2004.07.014 15577848

[6] Vignali DA Collison LW Workman CJ. How regulatory T cells work. Nat Rev Immunol. (2008) 8:523–32. 10.1038/nri2343 18566595 PMC2665249

[7] Sakaguchi S. Naturally arising CD4+ regulatory t cells for immunologic self-tolerance and negative control of immune responses. Annu Rev Immunol. (2004) 22:531–62. 10.1146/annurev.immunol.21.120601.141122 15032588

[8] Provoost S Maes T van Durme YM Gevaert P Bachert C Schmidt-Weber CB. Decreased FOXP3 protein expression in patients with asthma. Allergy (2009) 64:1539–46. 10.1111/j.1398-9995.2009.02056.x 19392991

[9] Bennett CL Christie J Ramsdell F Brunkow ME Ferguson PJ Whitesell L. The immune dysregulation, polyendocrinopathy, enteropathy, X-linked syndrome (IPEX) is caused by mutations of FOXP3. Nat Genet. (2001) 27:20–1. 10.1038/83713 11137993

[10] Jang M Cai L Udeani GO Slowing KV Thomas CF Beecher CW. Cancer chemopreventive activity of resveratrol, a natural product derived from grapes. Science (1997) 275:218–20. 10.1126/science.275.5297.218 8985016

[11] Cottrez F Hurst SD Coffman RL Groux H. T regulatory cells 1 inhibit a Th2-specific response *in vivo*. J Immunol, (2000) 165:4848–53. 10.4049/jimmunol.165.9.4848 11046008

[12] Zhang L Zhang Y Desrosiers M Wang C Zhao Y Han D. Genetic association study of FOXP3 polymorphisms in allergic rhinitis in a Chinese population. Hum Immunol. (2009) 70:930–4. 10.1016/j.humimm.2009.08.001 19679154

[13] Bottema RW Kerkhof M Reijmerink NE Koppelman GH Thijs C Stelma FF. X-chromosome Forkhead Box P3 polymorphisms associate with atopy in girls in three Dutch birth cohorts. Allergy (2010) 65:865–74. 10.1111/j.1398-9995.2009.02291.x 20028375

[14] Fodor E Garaczi E Polyánka H Koreck A Kemény L Széll M. The rs3761548 polymorphism of FOXP3 is a protective genetic factor against allergic rhinitis in the Hungarian female population. Hum Immunol. (2011) 72:926–9. 10.1016/j.humimm.2011.06.011 21763379

[15] Lin W Truong N Grossman WJ Haribhai D Williams CB Wang J. Allergic dysregulation and hyperimmunoglobulinemia E in Foxp3 mutant mice. J Allergy Clin Immunol. (2005) 116:1106–15. 10.1016/j.jaci.2005.08.046 16275384

[16] Solberg OD Ostrin EJ Love MI Peng JC Bhakta NR Hou L. Airway epithelial miRNA expression is altered in asthma. Am J Respir Crit Care Med, (2012) 186:965–74. 10.1164/rccm.201201-0027OC PMC3530212 22955319

[17] Singh NP Hegde VL Hofseth LJ Nagarkatti M Nagarkatti P. Resveratrol (trans-3,5,4'-trihydroxystilbene) ameliorates experimental allergic encephalomyelitis, primarily via induction of apoptosis in T cells involving activation of aryl hydrocarbon receptor and estrogen receptor. Mol Pharmacol. (2007) 72:1508–21. 10.1124/mol.107.038984 17872969 PMC4796949

[18] Guan H Singh NP Singh UP Nagarkatti PS Nagarkatti M. Resveratrol prevents endothelial cells injury in high-dose interleukin-2 therapy against melanoma. PLoS One, (2012) 7:e3(5650). 10.1371/journal.pone.0035650 PMC3331985 22532866

[19] Donnelly LE Newton R Kennedy GE Fenwick PS Leung RH Ito K. Anti-inflammatory effects of resveratrol in lung epithelial cells: molecular mechanisms. Am J Physiol Lung Cell Mol Physiol. (2004) 287:L774–83. 10.1152/ajplung.00110.2004 15180920

[20] Bradamante S Barenghi L Villa A. Cardiovascular protective effects of resveratrol. Cardiovasc Drug Rev. (2004) 22:169–88. 10.1111/j.1527-3466.2004.tb00139.x 15492766

[21] Bateman ED Hurd SS Barnes PJ Bousquet J Drazen JM FitzGerald JM. Global strategy for asthma management and prevention: GINA executive summary. Eur Respir J. (2008) 31:143–78. 10.1183/09031936.00138707 18166595

[22] Ortega H Llanos JP Lafeuille MH Duh MS Germain G Lejeune D. Effects of systemic corticosteroids on blood eosinophil counts in asthma: real-world data. J Asthma (2018):1–26. 10.1080/02770903.2018.1502301 30130418

[23] Durrani SR Viswanathan RK Busse WW. What effect does asthma treatment have on airway remodeling? Current perspectives. J Allergy Clin Immunol. (2011) 128:439–48; quiz 449-50. 10.1016/j.jaci.2011.06.002 21752441

[24] Royce SG Tang ML. The effects of current therapies on airway remodeling in asthma and new possibilities for treatment and prevention. Curr Mol Pharmacol. (2009) 2:169–81. 10.2174/1874467210902020169 20021456

[25] Shi Y Zhou J Jiang B Miao M. Resveratrol and inflammatory bowel disease. Ann N Y Acad Sci. (2017) 1403:38–47. 10.1111/nyas.13426 28945937

[26] Ma C Wang Y Dong L Li M Cai W. Anti-inflammatory effect of resveratrol through the suppression of NF-kappaB and JAK/STAT signaling pathways. Acta Biochim Biophys Sin. (2015) 47:207–13. 10.1093/abbs/gmu135 25651848

[27] Svajger U Jeras M. Anti-inflammatory effects of resveratrol and its potential use in therapy of immune-mediated diseases. Int Rev Immunol. (2012) 31:202–22. 10.3109/08830185.2012.665108 22587021

[28] Lee HY Kim IK Yoon HK Kwon SS Rhee CK Lee SY. Inhibitory effects of resveratrol on airway remodeling by transforming growth factor-beta/smad signaling pathway in chronic asthma model. Allergy Asthma Immunol Res. (2017) 9:25–34. 10.4168/aair.2017.9.1.25 27826959 PMC5102832

[29] Royce SG Dang W Yuan G Tran J El Osta A Karagiannis TC. Resveratrol has protective effects against airway remodeling and airway hyperreactivity in a murine model of allergic airways disease. Pathobiol Aging Age Relat Dis. (2011) 1:10.3402/PBA.v1i0.7134. 10.3402/PBA.v1i0.7134 22953028 PMC3417665

[30] Takeda M Tanabe M Ito W Ueki S Konnno Y Chihara M. Gender difference in allergic airway remodelling and immunoglobulin production in mouse model of asthma. Respirology (2013) 18:797–806. 10.1111/resp.12078 23490273

[31] Melgert BN Postma DS Kuipers I Geerlings M Luinge MA van der Strate BW. Female mice are more susceptible to the development of allergic airway inflammation than male mice. Clin Exp Allergy. (2005) 35:1496–503. 10.1111/j.1365-2222.2005.02362.x 16297148

[32] Fuseini H Newcomb DC. Mechanisms driving gender differences in asthma. Curr Allergy Asthma Rep. (2017) 17:19. 10.1007/s11882-017-0686-1 28332107 PMC5629917

[33] Shi JP Li XN Zhang XY Du B Jiang WZ Liu MY. Gpr97 is dispensable for inflammation in OVA-induced asthmatic mice. PLoS ONE (2015) 10:e0131461 10.1371/journal.pone.0131461 26132811 PMC4489018

[34] Chen L Yang S Zumbrun EE Guan H Nagarkatti PS Nagarkatti M. Resveratrol attenuates lipopolysaccharide-induced acute kidney injury by suppressing inflammation driven by macrophages. Mol Nutr Food Res. (2015) 59:853–64. 10.1002/mnfr.201400819 25643926 PMC4420731

[35] Altamemi I Murphy EA Catroppo JF Zumbrun EE Zhang J McClellan JL. Role of microRNAs in resveratrol-mediated mitigation of colitis-associated tumorigenesis in Apc(Min/+) mice. J Pharmacol Exp Ther. (2014) 350:99–109. 10.1124/jpet.114.213306 24817032 PMC4056272

[36] Rieder SA Nagarkatti P Nagarkatti M Multiple anti-inflammatory pathways triggered by resveratrol lead to amelioration of staphylococcal enterotoxin B-induced lung injury. Br J Pharmacol. (2012) 167:1244–58. 10.1111/j.1476-5381.2012.02063.x 22646800 PMC3504991

[37] Rao R Rieder SA Nagarkatti P Nagarkatti M. Staphylococcal enterotoxin B-induced microRNA-155 targets SOCS1 to promote acute inflammatory lung injury. Infect Immun. (2014) 82:2971–9. 10.1128/IAI.01666-14 24778118 PMC4097622

[38] Gadjeva M Paradis-Bleau C Priebe GP Fichorova R Pier GB. Caveolin-1 modifies the immunity to *Pseudomonas aeruginosa*. J Immunol. (2010) 184:296–302. 10.4049/jimmunol.0900604 19949109 PMC2900931

[39] Alghetaa H Mohammed A Sultan M Busbee P Murphy A Chatterjee S. Resveratrol protects mice against SEB-induced acute lung injury and mortality by miR-193a modulation that targets TGF-beta signalling. J Cell Mol Med. (2018) 22:2644–55. 10.1111/jcmm.13542 29512867 PMC5908132

[40] Rao R Nagarkatti P Nagarkatti M. Role of miRNA in the regulation of inflammatory genes in staphylococcal enterotoxin B-induced acute inflammatory lung injury and mortality. Toxicol Sci. (2015) 144:284–97. 10.1093/toxsci/kfu315 25564423 PMC4372662

[41] Pilbrow AP Ellmers LJ Black MA Moravec CS Sweet WE Troughton RW. Genomic selection of reference genes for real-time PCR in human myocardium. BMC Med Genomics (2008) 1:64. 10.1186/1755-8794-1-64 19114010 PMC2632664

[42] Ling H Wu Q Guo J Xu L Que Y. Comprehensive selection of reference genes for gene expression normalization in sugarcane by real time quantitative rt-PCR. PLoS ONE (2014) 9:e97469 10.1371/journal.pone.0097469 24823940 PMC4019594

[43] Kasimanickam V Kastelic J. Circulating cell-free mature microRNAs and their target gene prediction in bovine metritis. Sci Rep. (2016) 6:29509 10.1038/srep29509 27404038 PMC4941693

[44] Rajasingh J Lambers E Hamada H Bord E Thorne T. Cell-free embryonic stem cell extract-mediated derivation of multipotent stem cells from NIH3T3 fibroblasts for functional and anatomical ischemic tissue repair. Circ Res. (2008) 102:e107–17. 10.1161/CIRCRESAHA.108.176115 18483406 PMC2435186

[45] Price MM Oskeritzian CA Falanga YT Harikumar KB Allegood JC Alvarez SE. A specific sphingosine kinase 1 inhibitor attenuates airway hyperresponsiveness and inflammation in a mast cell-dependent murine model of allergic asthma. J Allergy Clin Immunol. (2013) 131:501–11 e1. 10.1016/j.jaci.2012.07.014 22939756 PMC3563730

[46] Crimi E Spanevello A Neri M Ind PW Rossi GA Brusasco V. Dissociation between airway inflammation and airway hyperresponsiveness in allergic asthma. Am J Respir Crit Care Med. (1998) 157:4–9. 10.1164/ajrccm.157.1.9703002 9445270

[47] Endo Y Nakayama T. Pathogenic Th2 (Tpath2) cells in airway inflammation. Oncotarget (2015) 6:32303–4. 10.18632/oncotarget.6033 26459386 PMC4741689

[48] Dahl R. Systemic side effects of inhaled corticosteroids in patients with asthma. Respir Med. (2006) 100:1307–17. 10.1016/j.rmed.2005.11.020 16412623

[49] Busse W Corren J Lanier BQ McAlary M Fowler-Taylor A Cioppa GD. Omalizumab, anti-IgE recombinant humanized monoclonal antibody, for the treatment of severe allergic asthma. J Allergy Clin Immunol. (2001) 108:184–90. 10.1067/mai.2001.117880 11496232

[50] Cave A Arlett P Lee E. Inhaled and nasal corticosteroids: factors affecting the risks of systemic adverse effects. Pharmacol Ther. (1999) 83:153–79. 10.1016/S0163-7258(99)00019-4 10576291

[51] Aich J Mabalirajan U Ahmad T Khanna K Rehman R Agrawal A. Resveratrol attenuates experimental allergic asthma in mice by restoring inositol polyphosphate 4 phosphatase (INPP4A). Int Immunopharmacol. (2012) 14:438–43. 10.1016/j.intimp.2012.08.017 22986054

[52] Liu Y Ma W Zhang P He S Huang D. Effect of resveratrol on blood pressure: a meta-analysis of randomized controlled trials. Clin Nutr. (2015) 34:27–34. 10.1016/j.clnu.2014.03.009 24731650

[53] Sahebkar A Serban C Ursoniu S Wong ND Muntner P Graham IM. Lack of efficacy of resveratrol on C-reactive protein and selected cardiovascular risk factors–Results from a systematic review and meta-analysis of randomized controlled trials. Int J Cardiol. (2015) 189:47–55. 10.1016/j.ijcard.2015.04.008 25885871

[54] Zhu X Wu C Qiu S Yuan X Li L. Effects of resveratrol on glucose control and insulin sensitivity in subjects with type 2 diabetes: systematic review and meta-analysis. Nutr Metab. (2017) 14:60. 10.1186/s12986-017-0217-z 29018489 PMC5610395

[55] Chen J Zhou H Wang J Zhang B Liu F Huang J. Therapeutic effects of resveratrol in a mouse model of HDM-induced allergic asthma. Int Immunopharmacol. (2015) 25:43–8. 10.1016/j.intimp.2015.01.013 25617148

[56] Lee M Kim S Kwon OK Oh SR Lee HK Ahn K. Anti-inflammatory and anti-asthmatic effects of resveratrol, a polyphenolic stilbene, in a mouse model of allergic asthma. Int Immunopharmacol. (2009) 9:418–24. 10.1016/j.intimp.2009.01.005 19185061

[57] Chung KF Barnes PJ. Cytokines in asthma. Thorax (1999) 54:825–57. 10.1136/thx.54.9.825 10456976 PMC1745579

[58] Kips JC. Cytokines in asthma. Eur Respir J. (2001) (Suppl. 34):24s–33s. 10.1183/09031936.01.00229601 12392032

[59] Chung F. Anti-inflammatory cytokines in asthma and allergy: interleukin-10, interleukin-12, interferon-gamma. Mediators Inflamm. (2001) 10:51–9. 10.1080/09629350120054518 11405550 PMC1781697

[60] Ogawa Y Duru EA Ameredes BT. Role of IL-10 in the resolution of airway inflammation. Curr Mol Med. (2008) 8:437–45. 10.2174/156652408785160907 18691071 PMC9159958

[61] Taganov KD Boldin MP Chang KJ Baltimore D. NF-kappaB-dependent induction of microRNA miR-146, an inhibitor targeted to signaling proteins of innate immune responses. Proc Natl Acad Sci USA. (2006) 103:12481–6. 10.1073/pnas.0605298103 16885212 PMC1567904

[62] Sheedy FJ Palsson-McDermott E Hennessy EJ Martin C O'Leary JJ Ruan Q. Negative regulation of TLR4 via targeting of the proinflammatory tumor suppressor PDCD4 by the microRNA miR-21. Nat Immunol. (2010) 11:141–7. 10.1038/ni.1828 19946272

[63] Bazzoni F Rossato M Fabbri M Gaudiosi D Mirolo M Mori L. Induction and regulatory function of miR-9 in human monocytes and neutrophils exposed to proinflammatory signals. Proc Natl Acad Sci USA. (2009) 106:5282–7. 10.1073/pnas.0810909106 19289835 PMC2664036

[64] Liu G Friggeri A Yang Y Park YJ Tsuruta Y Abraham E. miR-147, a microRNA that is induced upon Toll-like receptor stimulation, regulates murine macrophage inflammatory responses. Proc Natl Acad Sci USA. (2009) 106:15819–24. 10.1073/pnas.0901216106 19721002 PMC2747202

[65] Du C Liu C Kang J Zhao G Ye Z Huang S. MicroRNA miR-326 regulates TH-17 differentiation and is associated with the pathogenesis of multiple sclerosis. Nat Immunol. (2009) 10:1252–9. 10.1038/ni.1798 19838199

[66] Zheng Y Josefowicz SZ Kas A Chu TT Gavin MA Rudensky AY. Genome-wide analysis of Foxp3 target genes in developing and mature regulatory T cells. Nature (2007) 445:936–40. 10.1038/nature05563 17237761

[67] Tan LP Wang M Robertus JL Schakel RN Gibcus JH Diepstra A. miRNA profiling of B-cell subsets: specific miRNA profile for germinal center B cells with variation between centroblasts and centrocytes. Lab Invest. (2009) 89:708–16. 10.1038/labinvest.2009.26 19349957

[68] Basso K Sumazin P Morozov P Schneider C Maute RL Kitagawa Y. Identification of the human mature B cell miRNome. Immunity (2009) 30:744–52. 10.1016/j.immuni.2009.03.017 19446474 PMC2764486

[69] Xiao C Rajewsky K. MicroRNA control in the immune system: basic principles. Cell (2009) 136:26–36. 10.1016/j.cell.2008.12.027 19135886

[70] Bae S Lee EM Cha HJ Kim K Yoon Y Lee H. Resveratrol alters microRNA expression profiles in A549 human non-small cell lung cancer cells. Mol Cells (2011) 32:243–9. 10.1007/s10059-011-1037-z 21887509 PMC3887628

[71] Tili E Michaille JJ Alder H Volinia S Delmas D Latruffe N. Resveratrol modulates the levels of microRNAs targeting genes encoding tumor-suppressors and effectors of TGFbeta signaling pathway in SW480 cells. Biochem Pharmacol. (2010) 80:2057–65. 10.1016/j.bcp.2010.07.003 20637737 PMC3918904

[72] Venkatadri R Muni T Iyer AK Yakisich JS Azad N. Role of apoptosis-related miRNAs in resveratrol-induced breast cancer cell death. Cell Death Dis. (2016) 7:e2104 10.1038/cddis.2016.6 26890143 PMC5399194

[73] Shukla Y Singh R. Resveratrol and cellular mechanisms of cancer prevention. Ann N Y Acad Sci. (2011) 1215:1–8. 10.1111/j.1749-6632.2010.05870.x 21261635

[74] Sonkoly E Pivarcsi A. microRNAs in inflammation. Int Rev Immunol. (2009) 28:535–61. 10.3109/08830180903208303 19954362

[75] Singh RP Massachi I Manickavel S Singh S Rao NP Hasan S. The role of miRNA in inflammation and autoimmunity. Autoimmun Rev. (2013) 12:1160–5. 10.1016/j.autrev.2013.07.003 23860189

[76] Sonkoly E Stahle M Pivarcsi A. MicroRNAs and immunity: novel players in the regulation of normal immune function and inflammation. Semin Cancer Biol. (2008) 18:131–40. 10.1016/j.semcancer.2008.01.005 18291670

[77] Hippen KL Loschi M Nicholls J MacDonald KPA Blazar BR. Effects of microRNA on regulatory T cells and implications for adoptive cellular therapy to ameliorate graft-versus-host disease. Front Immunol. (2018) 9:57. 10.3389/fimmu.2018.00057 29445371 PMC5797736

[78] Chong MM Rasmussen JP Rudensky AY Littman DR. The RNAseIII enzyme Drosha is critical in T cells for preventing lethal inflammatory disease. J Exp Med. (2008) 205:2005–17. 10.1084/jem.20071219090508c 18725527 PMC2526196

[79] Lu LF Thai TH Calado DP Chaudhry A Kubo M Tanaka K. Foxp3-dependent microRNA155 confers competitive fitness to regulatory T cells by targeting SOCS1 protein. Immunity (2009) 30:80–91. 10.1016/j.immuni.2008.11.010 19144316 PMC2654249

[80] He M Song G Yu Y Jin Q Bian Z. LPS-miR-34a-CCL22 axis contributes to regulatory T cell recruitment in periapical lesions. Biochem Biophys Res Commun. (2015) 460:733–40. 10.1016/j.bbrc.2015.03.098 25817785

[81] Al-Alawi M Hassan T Chotirmall SH. Transforming growth factor beta and severe asthma: a perfect storm. Respir Med. (2014) 108:1409–23. 10.1016/j.rmed.2014.08.008 25240764

[82] Wan YY Flavell RA. ‘Yin-Yang' functions of transforming growth factor-beta and T regulatory cells in immune regulation. Immunol Rev. (2007) 220:199–213. 17979848 10.1111/j.1600-065X.2007.00565.x PMC2614905

