# Corrigendum: Resveratrol attenuates allergic asthma and associated inflammation in the lungs through regulation of miRNA-34a that targets FoxP3 in mice

**DOI:** 10.3389/fimmu.2023.1130947

**Published:** 2023-02-14

**Authors:** Esraah Alharris, Hasan Alghetaa, Ratanesh Seth, Saurabh Chatterjee, Narendra P. Singh, Mitzi Nagarkatti, Prakash Nagarkatti

**Affiliations:** ^1^ Department of Pathology, Microbiology and Immunology, School of Medicine, University of South Carolina, Columbia, SC, United States; ^2^ Environmental Health and Disease Laboratory, Department of Environmental Health Sciences, Arnold School of Public Health, University of South Carolina, Columbia, SC, United States

**Keywords:** Asthma, resveratrol, miRNA-34a, Foxp3, T regulatory cells

In the published article, there was an error in **Figure 5B**, Ova-veh 20X panel, as published. The wrong microscopy picture of immunohistochemistry was provided. The corrected **Figure 5** and its caption appear below.

**Figure 5:**
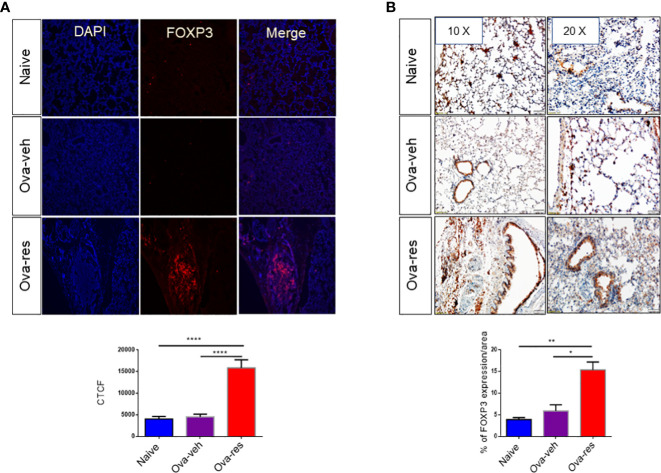
Resveratrol treatment leads to induction of FOXP3+ cells in the lungs: Immunofluorescence and immunohistochemistry were performed to determine the expression of FOXP3 in lung tissues and FoxP3 expression in the cells was assessed using corrected total cell fluorescence (CTCF) and ImageJ software. **(A)** Shows the expression of FOXP3 in lung tissues. The data in vertical bars represent Mean+/– SEM of 10 random spots analyzed. Significance (*p < 0.05) of FoxP3 expression between the groups was analyzed using Student’s t-test. **(B)** Shows FoxP3 expression in lung tissues by performing immunohistochemistry. The data represented as Mean+/– SEM of random 3–5 spots that were analyzed. The number of mice used (Naïve: n = 3, OVA-veh: n = 3, and OVA-res: n = 3). Significance (**p <0.01, ****p <0.0001) in FoxP3 expression was detected using one-way ANOVA and *post-hoc* Tukey’s test.

The authors apologize for this error and state that this does not change the scientific conclusions of the article in any way. The original article has been updated.

