# Supplementary material for: Resveratrol Attenuates Allergic Asthma and Associated Inflammation in the Lungs Through Regulation of miRNA-34a That Targets FoxP3 in Mice
Source: Front Immunol. 2018 Dec 20;9:2992. doi: 10.3389/fimmu.2018.02992 (PMC6306424; doi:10.3389/fimmu.2018.02992)
Supplement: Supplementary file 1 [file Table_1.DOCX]

Supplementary table.1. Primer sequences for qPCR analysis.

| *FOXP3* (Forward) | 5`-CCCATCCCCAGGAGTCTTG-3` |
| --- | --- |
| *FOXP3* (Reverse) | 5`-ACCATGACTAGGGGCACTGTA-3` |
| *IL-13* (Forward) | 5`-CCTGGCTCTTGCTTGCCTT-3` |
| *IL-13* (Reverse) | 5`-GGTCTTGTGTGATGTTGCTCA-3` |
| *IL-10* (Forward) | 5`-GCTCTTACTGACTGGCATGAG-3` |
| *IL-10* (Reverse) | 5`-CGCAGCTCTAGGAGCATGTG-3` |
| *GATA3* (Forward) | 5`-CTCGGCCATTCGTACATGGAA-3` |
| *GATA3* (Reverse) | 5`-GGATACCTCTGCACCGTAGC-3` |
| *GAPDH* (Forward) | 5`-AGGTCGGTGTGAACGGATTTG-3` |
| *GAPDH* (Reverse) | 5`-TGTAGACCATGTAGTTGAGGTCA-3` |
| Mmu-miR-34a-5p | 5'-UGGCAGUGUCUUAGCUGGUUGU-3` |
